# Voice selectivity in the temporal voice area despite matched low-level acoustic cues

**DOI:** 10.1038/s41598-017-11684-1

**Published:** 2017-09-14

**Authors:** Trevor R. Agus, Sébastien Paquette, Clara Suied, Daniel Pressnitzer, Pascal Belin

**Affiliations:** 1 SARC, School of Arts, English and Languages, Queen’s University, Belfast, BT7 1NN UK; 2 Department of Psychology, International Laboratory for Brain Music and Sound Research, Center for Research on Brain, Language and Music, University of Montreal, Montreal, (QC) H3C 3J7 Canada; 3 Département Action et Cognition en Situation Opérationnelle, Institut de recherche biomédicale des armées, 91223 Brétigny sur Orge, France; 4 Laboratoire des Systèmes Perceptifs, UMR 8248, CNRS and Ecole normale supérieure, 75005 Paris, France; 5 Voice Neurocognition Laboratory, Institute of Neuroscience and Psychology, University of Glasgow, Glasgow, G12 8QQ UK; 6 Institut des Neurosciences de La Timone, Aix-Marseille Université, 13005 Marseille, France

## Abstract

In human listeners, the temporal voice areas (TVAs) are regions of the superior temporal gyrus and sulcus that respond more to vocal sounds than a range of nonvocal control sounds, including scrambled voices, environmental noises, and animal cries. One interpretation of the TVA’s selectivity is based on low-level acoustic cues: compared to control sounds, vocal sounds may have stronger harmonic content or greater spectrotemporal complexity. Here, we show that the right TVA remains selective to the human voice even when accounting for a variety of acoustical cues. Using fMRI, single vowel stimuli were contrasted with single notes of musical instruments with balanced harmonic-to-noise ratios and pitches. We also used “auditory chimeras”, which preserved subsets of acoustical features of the vocal sounds. The right TVA was preferentially activated only for the natural human voice. In particular, the TVA did not respond more to artificial chimeras preserving the exact spectral profile of voices. Additional acoustic measures, including temporal modulations and spectral complexity, could not account for the increased activation. These observations rule out simple acoustical cues as a basis for voice selectivity in the TVAs.

## Introduction

**Figure 1:**
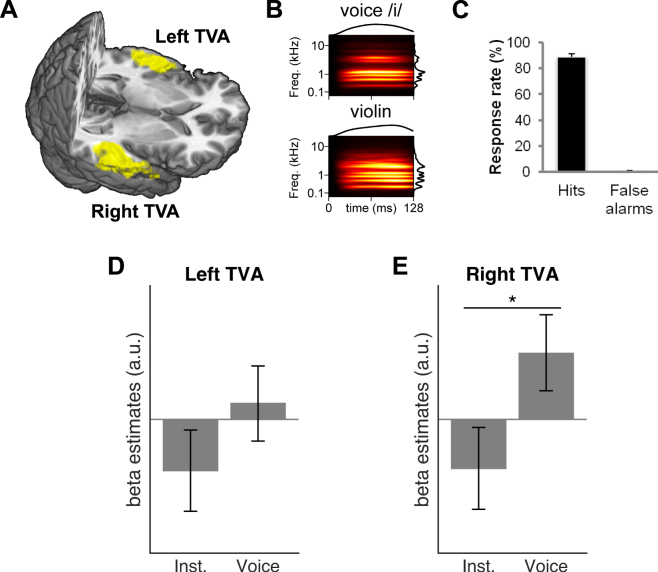
(**A**) The left and right TVAs, highlighted in yellow on a 3D rendering of a template brain, were identified using a“voice localizer” and the group-level contrast of vocal vs. nonvocal sounds (p < 0.05, FWE) (**B**) Auditory spectrograms^22^ of exemplars of the voice and instrument stimuli in Experiment 1, with amplitude envelopes over time (top panels) and auditory spectra (side panels). (**C**) Mean performance on the one-back task. (**D**) Mean and s.e.m. of parameter estimates in response to Voice and Instrument conditions in the left TVA. (**E**) As for panel D but for the right TVA, which shows greater activity to the voice stimuli.

Perceptual systems must transform the minutiae encoded by sensory receptors into broad and stable categories, upon which behavior can be based. For the auditory system, Belin and colleagues identified, using functional magnetic resonance imaging (fMRI), a region of the superior temporal sulcus (STS) selective to what is arguably the most behaviorally important sound category for human listeners: the human voice. In multiple studies, these regions of secondary auditory cortex showed a greater response to vocal sounds, including both speech and non-speech sounds, compared to a wide range of nonvocal control sounds, including scrambled voices, environmental noises, and even animal cries^1^. By analogy with selectivity to human faces in the visual modality, these regions became known as the temporal voice areas (TVAs; Fig. 1A). The TVAs have been observed in 7-month-old infants, suggesting an early development^2^ and homologues of the TVAs have been found in animals (e.g. macaques^3^).

How do the TVAs achieve their selectivity to vocal sounds? A parsimonious explanation is that the TVAs respond to acoustical cues specific to human voices, or at least, to cues that distinguish vocal sounds from the control sounds investigated so far. Indeed, the control sounds used for the standard “voice localizer” had been selected for maximal variety, but without explicit control for acoustical cues^1, 4^. It has since been noted that vocal sounds tend to have more harmonic structure than nonvocal sounds and that this could account for the cortical selectivity to speech sounds^5, 6^. Nonvocal sounds also tend to display less complexity, with simpler spectra or less amplitude variation over time^7^.

Here, we aimed at testing directly whether the TVA remained selective to the human voice even when a range of possible acoustic confounds were explicitly controlled for. In a first experiment, we contrasted single sung vowels with individual notes played by musical instruments. Both categories were exactly matched for pitch and harmonic-to-noise ratios^6^. Moreover, since all stimuli were brief harmonic sounds, both categories were of comparable spectrotemporal complexity^7^. We further controlled for complexity by computing indices of temporal and spectral modulation at the output of an auditory model^8, 9^. We also took into account a salient timbre dimension, brightness, which may play a role in natural sound representation^5^. In a second experiment, we used “auditory chimeras”^10^ to provide an even closer match between vocal and nonvocal control sounds. We used two types of chimera, which were formed with the spectral content of a voice but the temporal variations of an instrument, or vice versa. Thus, the two types of chimera together included all of the acoustical features of the voice, but each specific type only included a subset of those features. We tested whether we could observe selectivity for voice in the TVAs despite matching and controlling for multiple low-level acoustic cues.

## Materials and Methods

### Stimuli

In Experiment 1, there were two categories of stimuli, “vocal” and “instrumental”. Both sets of stimuli were taken from the RWC database^11^, as for related behavioral studies^10, 12^.

We computed several acoustic indices for the RWC sound set. First, the harmonic-to-noise ratio^6^ (HNR) was calculated using the Harmonicity object’s cross-correlation method in the Praat software (www.praat.org). A descriptor of spectral shape and indicator of brightness, the spectral centroid, was computed by estimating the center of gravity of the average pressure variance (mean of half-wave rectified signals, low-passed at 70 Hz) at the output of an auditory filterbank^13^. Finally, we used a signal-processing model of cortical processing^8^, to evaluate spectral scale and temporal rate. Scale is a measure of spectral shape, with fine spectral details associated with high scales. Rate is a measure of amplitude modulation, with high rates associated with faster fluctuations. We chose to summarize the output of the cortical model with two values, “dominant scale” and “dominant rate”, center of gravity of rate and scale, calculated as described by Joly *et al* ^9^. High rates or scales may be associated with higher complexity, as they indicate finer spectral details and faster temporal fluctuations. A combination of dominant rate and dominant scale has been shown to distinguish between speech utterances and scrambled or environmental sounds^9^.

Sounds were selected from the RWC database such that the pitch range and the mean harmonic-to-noise ratios (HNR) for the voices did not differ from those of the instruments. This resulted in the selection of the vowels /a/, /e/, /i/, and /o/ sung by two male and two female singers in a one-octave range from A3 to G#4 for the vocal stimuli, and of the notes in the same one-octave range played on 16 instruments (oboe, clarinet, bassoon, saxophone, trumpet, trombone, horn, guitar, mandolin, ukulele, harpsichord, piano, marimba, violin, viola, and cello) for the instrument stimuli. Sounds were faded out at 128 ms with a 5-ms raised-cosine window. All stimuli were normalized by their root-mean-square (RMS) to the same average intensity. The set was thus matched in terms of pitch range (A3 to G#4), duration (128 ms), RMS amplitude, and HNR (M = 24.1 dB, SD = 5.5 dB for the voices, *M* = 23.5 dB, SD = 3.5 dB for the instruments, one-way ANOVA *F*_1,30_ = 2.42, *p* = 0.13). Neither spectral centroid nor dominant rate differed significantly between voice and instrument stimuli (*p* ≥ 0.27), but voice stimuli had a slightly larger dominant scale than instrument stimuli (*M* = 1.37 v. 1.26 cycles per octave; *F*_1,30_ = 14.99, *p* < 0.001), corresponding to the voices having a slightly less complex spectrum on average.

In Experiment 2, auditory chimeras^10^ were formed by combining acoustic characteristics of voice (/a/ and /i/) and instrumental stimuli (violin and cello). The voice and instrument recordings were the same as those used in Experiment 1, but truncated at 250 ms. The construction of the chimeras is described in detail by Agus *et al* ^10^. In brief, both voice and instrumental stimuli were passed through an auditory filterbank^13^, simulating the frequency selectivity of the human ear. To generate an auditory chimera with the temporal structure of a string instrument and “auditory spectrum” of a voice, the levels of frequency bands of the string instrument were changed to the levels of the corresponding frequency bands for the voice, and all resulting bands were added together. The resulting auditory chimera has the temporal structure of the string instrument (including frequency-dependent envelopes, pitch instabilities, harmonic and noise components) but with the distinctive vowel-like spectral shape of the voice, including formants. Audio demonstrations of these chimeras are available at http://audition.ens.fr/chimeras. All pairwise chimeras were generated from four recorded sounds (/a/, /i/, violin, and cello) for each of 12 pitches (again A3 to G#4). This resulted in 16 chimeras (four vowels/instruments crossed pairwise with each of the four types of sound) at each of 12 notes covering an octave range, for a total of 192 stimuli.

### Procedure

In Experiment 1, an event-related design was used in which the voice and instrument stimuli were played in trials of six identical repetitions (mini-block duration: 768 ms). Trials were presented in pseudo-random order at a stimulus onset asynchrony (SOA) of 2 seconds. For each of the two categories, there were 16 different sounds at 12 different pitches. Each sound was presented on two different trials, resulting in 768 trials. Participants performed a one-back task, pressing a button when they heard a sound identical to the one in the immediately preceding trial. For this purpose, one in twenty trials were repeated, resulting in an additional 38 trials. Twenty-second pauses occurred every 290 seconds, forming six blocks. A total of 806 volumes were acquired for Experiment 1 within approximately 30 minutes. Stimuli were presented at a comfortable listening level over MRI-compatible electrostatic in-ear headphones (Sensimetrics Corporation, USA) using an M-Audio Audiophile 2496 soundcard. The response pad was an MRI-compatible response pad (Lumitouch).

In Experiment 2, participants were scanned while performing an active voice/instrument categorization task by pressing one of two buttons. Stimuli corresponding to four categories (Voice; Instrument; and two types of Voice-Instrument chimeras; cf. Stimuli) were presented in pseudo-random order with SOAs of 2 s. On each trial, the short stimulus was presented four times in quick succession, resulting in 1 s of sound per trial. The design followed a 2 × 2 factorial design with Temporal structure (T: Voice; Instrument) and auditory Spectrum (S: Voice; Instrument) as factors. Twenty-second pauses occurred every 296 seconds, forming three blocks. The 192 stimuli were presented twice to each participant. A total of 384 scans were acquired for Experiment 2, within approximately 13 minutes.

The third functional series consisted of a “voice localizer” scan to identify voice-selective cortex. Volumes consisting of 32 axial images (voxel size: 3 × 3 × 3 mm^3^) covering the whole brain were acquired with a TR of 2 seconds, while subjects listened passively to 8-s blocks of either vocal sounds, nonvocal sounds or silence, presented in an efficiency-optimized order at a 10-s SOA^1^. Finally, a T1-weighted anatomical scan was acquired (voxel size: 1 × 1 × 1 mm^3^).

The whole scanning session lasted approximately 75 minutes, including participants’ installation and pauses between runs. Matlab (Mathworks, Inc.) and the Psychophysics Toolbox^14^ were used for experimental control and data acquisition.

### Participants

Twenty-two neurotypical adult volunteers (11 M, 11 F) aged 18–33 years old (*M* = 25.5, *SD* = 4.3) participated in the study. Eighteen subjects were right-handed, two left-handed, and two ambidextrous. The ethical committee from the University of Glasgow approved the study. All experiments were performed in accordance with relevant guidelines and regulations. All volunteers provided informed written consent beforehand and received payment for participation.

### fMRI scanning

Images of cerebral structure and oxygenation level (BOLD) were acquired on a 3 T Tim Trio Scanner (Siemens) and a 32-channel head coil at the Centre for Cognitive Neuroimaging (CCNi) of the Institute of Neuroscience and Psychology, University of Glasgow. Three series of functional images were acquired using a T2*-weighted echo-planar-imaging (EPI) sequence. In the first two functional series, corresponding to Experiments 1 and 2, respectively, volumes consisting of 16 axial slices oriented parallel to the Sylvian fissure (voxel size: 2.5 × 2.5 × 2.5 mm^3^; gap: 15%) were acquired at a TR of 2 seconds.

### Analysis

#### MRI Preprocessing

Data analysis was performed using Statistical Parametric Mapping (SPM8; Wellcome Department of Cognitive Neurology). Images were realigned to correct head motion with the first volume of the first session as reference. T1-weighted structural images were co-registered to the mean image created by the realignment procedure and used for normalization of functional images onto the Montreal Neurological Institute (MNI) Atlas using normalization parameters derived from segmentation of the anatomical image. Finally, each image was smoothed with an isotropic 8-mm full-width-at-half-maximum Gaussian kernel.

#### General Linear Model

EPI time-series were analyzed using the general linear model (GLM) as implemented in SPM8. For each participant (1^st^-level analysis) the localizer and experimental runs were modeled separately. For the voice localizer, voice and nonvoice blocks were modeled as events using the canonical hemodynamic response function (HRF) with realignment parameters entered as parametric modulators. A “voice > non-voice” contrast was created for each subject and entered at the group level (2^nd^-level analysis) in a one-sampled *t*-test of their difference. Two regions of interest (ROIs) consisting of group-level voice-sensitive cortex in the left and right hemispheres were defined based on this contrast (TVA L and TVA R) at a threshold of *p* < 0.05 (FWE), using the MarsBaR ROI toolbox for SPM^15^. Analyses for Experiments 1 and 2 were conducted on voxels contained in these independently identified ROIs. For Experiment 1, a model consisting of three conditions (Voice; Instrument; sound repetitions and button presses) was generated. For each subject, the average values of the contrasts “Voice vs. baseline” and “Instruments vs. baseline” were computed and entered at the group-level in two, two-sample t-tests comparing responses to Voices vs. Instruments in the left and right TVAs, respectively. In a follow-up analysis, to control for acoustic differences, parametric modulators were added in the following order: pitch, HNR, dominant scale, dominant rate and spectral centroid. To test for similar effects in the primary auditory cortex (A1), the voice localizer was used again to define ROIs. The locations of the left and right A1 were estimated using the all-stimuli > silence contrast, identifying the auditory-sensitive cortices; 5-mm radius spheres were created around peak values in each hemisphere.

For Experiment 2, a model with four conditions was generated: Voice; Instrument; Voice-Instrument chimera; and Instrument-Voice chimera. For each subject, the ROI-average parameter estimates of each condition vs. baseline were entered in two repeated-measures ANOVAs with spectrum (Voice; Instrument) and temporal structure (Voice; Instrument) as factors, for the left and right TVA, respectively. The response to voice stimuli was also compared to the average of the three non-voice conditions (Instruments; and the two chimeras) via paired *t*-tests.

#### Behavioral analysis

Responses were associated with the stimulus whose onset was in the preceding 100–2100 ms. In Experiment 2, the analysis focused on the number of stimuli that were categorized as “voice”, not counting trials where both or neither of the buttons were pressed (0.3% and 1% of trials respectively). One listener’s results suggested they had used the buttons systematically with the opposite senses, so their interpretation was also reversed during the analysis.

## Results

### Voice localizer

The group-level contrast of activity elicited by vocal vs. nonvocal sounds during the voice-localizer scan highlighted the classical localization of the TVAs along the middle part of the superior temporal sulcus/gyrus bilaterally^1^, with group-level peaks at (60, −13, −2) and (−63, −28, 4). Voxels showing a significantly greater response to vocal vs. nonvocal sounds at a threshold of *p* < 0.05 (FWE) were included in two ROIs corresponding to the left and right TVAs, respectively (Fig. 1A).

### Experiment 1

In experiment 1, subjects were presented with brief sounds from two categories (voices and instruments) matched in pitch, duration, intensity, and harmonicity (Fig. 1B). In the right TVA, there was a significantly greater BOLD response to the voice stimuli than to the instrument stimuli (*t*_21_ = 2.54, *p* = 0.02; Fig. 1E). The contrast did not reach significance in the left TVA (*t*_21_ = 1.58, *p* = 0.13; Fig. 1D). No voice-sensitive regions other than the TVA were identified (FWE p < 0.05) using the (voice > non-voice) contrast. In a follow-up analysis, where the acoustic parameters of pitch, HNR, dominant scale, dominant rate and spectral centroid were entered as auditory parametric modulators, the right TVA response remained (*t*_21_ = 3.38, *p* = 0.03). The high performance on an orthogonal one-back task (Fig. 1C) confirmed that listeners had attended to the sounds.

### Experiment 2

**Figure 2:**
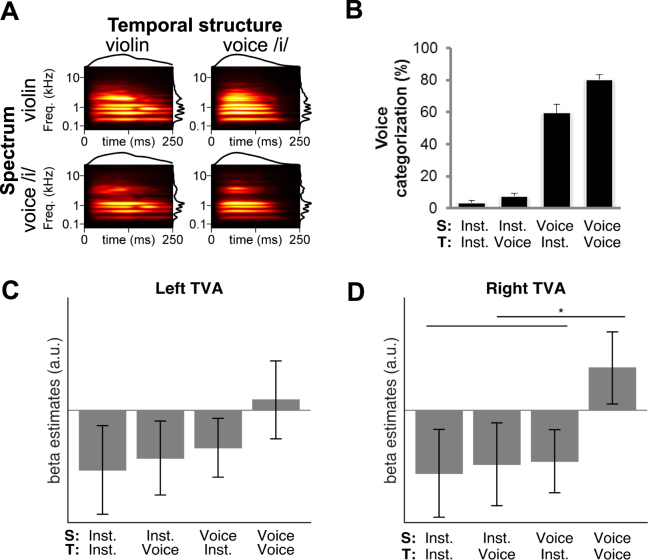
(**A**) As for panel B, but for exemplars of the stimuli used in Experiment 2, organized in a 2 × 2 factorial design with temporal structure (T: Voice; Instrument) and auditory spectrum (S: Voice; Instrument) as factors. The top left (Instrument) and bottom right (Voice) stimuli correspond to natural categories, while the other two stimuli correspond to “chimeras”. (**B**) Average behavioral categorization as voice of the four stimulus categories. (**C**) Mean and s.e.m. of parameter estimates in response to the four stimulus conditions in the left TVA. (**D**) As for panel C, but for the right TVA, in which the response to Voice is significantly greater than for the other three stimulus categories. *p < 0.05.

In Experiment 2, participants performed a voice/instrument categorization task on stimuli from four categories: voices; instruments; and two categories of voice-instrument “chimeras”, each with the spectrum of one sound and the temporal structure of the other one (see Fig. 2A). The right TVA was selective to purely vocal stimuli relative to the average of the three types of instrumental or vocal-instrumental chimeras (*t*_21_ = 2.16, *p* = 0.04; Fig. 2D). We found no significant effect of auditory spectrum (*F*_1,21_ = 1.75, *p* = 0.20) nor any interaction between auditory spectrum and temporal structure (*F*_1,21_ = 0.63, *p* = 0.44) in the right TVA. In the left TVA (Fig. 2C), no significant main effect of auditory spectrum (*F*_1,21_ = 0.90, p = 0.35) or TS (*F*_1,21_ = 0.74, *p* = 0.40) was observed, nor any interaction between auditory spectrum and temporal structure (*F*_1,21_ = 0.15, *p* = 0.71). The comparison of purely vocal chimeras to the average of the three other chimeras showed no significant difference (*t*_21_ = 1.23, *p* = 0.23) in the left TVA.

The proportion of trials that were categorized as voices varied between the four categories of stimulus (*F*_1.59,33.4_ = 218.68, *p* < 0.001 with a Greenhouse-Geisser correction). Pairwise comparisons were all highly significant (*t*_21_ ≥ 3.02, *p* ≤ 0.006). Importantly, these behavioral responses differed from the patterns of neural activations (Fig. 2B). The chimeras with the voice auditory spectra were categorized as vocal stimuli more often (*M* = 59%) than either the intact string stimuli or the chimera with the vocal temporal structure (*M* = 8% and 4% respectively). However, as reported above, there was no effect of auditory spectrum in either TVA. In addition, there were no significant correlations between behavioral responses and either left or right TVA fMRI responses across participants and within stimulus type (|*r*| ≤ 0.32 in 2 × 4 comparisons). Pooling responses from both types of chimeric stimuli did not yield any significant correlations either (|*r*| ≤ 0.07). Note that for the chimeras, there was no ground truth as to whether they should be categorized as voices or instruments, so variability in behavioral responses may have been determined primarily by how listeners interpreted the question. Importantly, both observations show that the TVA activity was not reflecting the reported decisions of listeners during the task, but rather, the presence of a natural vocal stimulus.

### Responses in A1

In a post hoc analysis in a ROI defined around A1 (see Methods), we observed significantly stronger responses to vocal sounds in both hemispheres (both *p* < 0.02) for Experiment 1. However, no significant differences between vocal and chimeric stimuli were observed around A1 for Experiment 2. In particular, the contrast between purely vocal chimeras to the average of the three other chimeras showed no significant differences (*p* > 0.05), in contrast to the specific difference for the same contrast in the right TVA.

## Discussion

We contrasted the brain activity elicited by sounds from the human voice with that elicited by musical instrument sounds, both categories having been deliberately matched for pitch, duration, intensity, and, importantly, harmonic-to-noise ratio. We observed a preferential response of the right-TVA ROI to vocal sounds, even after these and additional acoustic cues were entered as parametric modulators in the fMRI analysis (pitch, HNR, spectral scale, temporal rate, spectral centroid). In a second experiment, we used chimeras to present sub-sets of vocal features. Only the pure vocal stimuli induced a preferential response of the right TVA. Noticeably, even chimeras that were behaviorally categorized as voices did not cause such neural responses.

In addition to selectivity to vocal sounds in the TVA, we also observed some selectivity to vocal sounds in primary auditory cortex for Experiment 1. Such effects can be noted in previous data for vocal sounds^1^ or even speech sounds^16^, in addition to the more prominent activations in non-primary areas. In our case, even though we matched pitch, HNR, duration and RMS, other acoustic features represented in A1 may have partially distinguished between vocal and non-vocal sounds. However, this selectivity in A1 was not observed with similar sounds in Expt 2. These results are consistent with selectivity to vocal sounds being progressively refined along the auditory pathways, up to the TVA.

Given our statistical criterion, we observed selectivity for voices only in the right TVA, and not in the left TVA. This novel observation, for carefully matched stimuli, is consistent with previous results showing greater activation in the right hemisphere using more diverse stimuli^1^. The use of particularly short vocal stimuli (128 or 250 ms) may also have reduced the effect size of the BOLD response, and possibly underestimated the selectivity observed in the left TVA, in which a trend was present, but did not reach significance. Although our short stimuli were repeatedly presented to try and elicit more robust responses, this may have induced repetition-suppression^17^ and thus weaker responses than could have been obtained with more variable stimuli, such as those of the standard voice localizer. This conservative choice was motivated by the aim to have as much control as possible of acoustic cues, while allowing a direct comparison with previous human behavioral data showing a voice advantage^10^. Future experiments could contrast longer stimuli such as musical melodies of either instrument sounds or sung vowels, to minimize the effects of repetition-suppression.

Does this mean that the only genuine vocal selectivity is in the right TVA? By impoverishing the stimuli in order to control for acoustic cues, it could be argued that we were not investigating representative sets of vocal versus nonvocal sounds. The criticism certainly holds, and the additional selectivity to voices in the left TVA, even if it could be accounted for by simple acoustic cues, may still be functionally useful. Nevertheless, it is noticeable that the activation observed in the right TVA closely reflected behavioral processing efficacy with the same sounds in other studies: we previously found that listeners were more accurate^10, 12^ and their speeded responses were faster^10^ for vocal sounds relative to matched musical instrument sounds. Furthermore, faster responses were observed for intact vocal stimuli only, and not for chimeras, again precisely reflecting the response of the right TVA observed here. This suggests that the remaining selectivity we documented in the right TVA is functionally meaningful.

Our results rule out some *a priori* plausible acoustic cues for the selectivity to voices observed in the right TVA. Although harmonicity contributes to the preferential response to voices^5, 6^, it did not account entirely for the selective response. The dominant spectral scale and temporal rates can distinguish speech sounds from environmental sounds^9^ but, when entered as modulators, they could not account for the vocal selectivity either. It is, of course, possible that we did not identify a crucial acoustic cue unique to vocal sounds. Indeed, it is impossible to match perfectly all possible acoustic cues between two sound categories, as a perfect match would logically imply identical sounds. Thus, for the first experiment, we selected a range of plausible acoustic cues based on previous investigations^5–7, 9^. Of note, however, is that for the second experiment, the chimeras presented *all possible* spectral and temporal cues to our vocal sound set, split over complementary subsets for the different chimeras. So, the cues involved need to be complex and perhaps consist of a conjunction of simpler cues.

Another possibility was that the TVA response was not directly related to the acoustics of the sounds, but rather, reflected the behavioral decision to categorize a sound as a voice. The neural computation subserving the behavioral decision could be performed elsewhere in the brain, for instance through distributed processing^18^. Our results cannot be explained purely in terms of decisional outcome: the one-back task of the first experiment was not biased towards either of the stimulus categories, and the subjective categorizations in the second experiment did not match neural response patterns in the right TVA. Its responses could have reflected the behavioral value, such as the “naturalness” of the stimuli^19^ or more emotional responses to the voice mediated by the amygdala^4^, but the question remains as to how naturalness or emotional values are related to acoustical cues.

We propose an interpretation of TVA processing in terms of selectivity to complex cues or cue conjunctions. These complex selectivities^20^ could be built up from simpler neural receptive fields along the auditory hierarchy, as the information to select voices has been shown to be available in distributed codes^18^. The further processing in the TVAs may then subserve the remarkably efficient processing of vocal sounds by human listeners^10^. The exact nature of the complex cues selected by the TVAs remains to be described. An intriguing possibility is that there may not be a unique, hard-wired set of features to detect voices^21^. Rather, a gradual refinement of the acoustical features used to detect the voice, through personal experience, would be consistent with the large inter-subject variability observed when mapping voice selectivity across large cohorts of listeners^4^.
